# Diversity of ESI-MS Based Phosphatidylcholine Profiles in Basidiomycetes

**DOI:** 10.3390/jof8020177

**Published:** 2022-02-11

**Authors:** Ekaterina R. Kotlova, Svetlana V. Senik, Bairta S. Manzhieva, Anna A. Kiyashko, Natalia V. Shakhova, Roman K. Puzansky, Sergei V. Volobuev, Alexander D. Misharev, Eugeny B. Serebryakov, Nadezhda V. Psurtseva

**Affiliations:** 1 Komarov Botanical Institute, Russian Academy of Sciences RAS, 197376 Saint-Petersburg, Russia; senik@binran.ru (S.V.S.); bmanzhieva@binran.ru (B.S.M.); Anna.Kiyashko@binran.ru (A.A.K.); NShakhova@binran.ru (N.V.S.); puzansky@yandex.ru (R.K.P.); sergvolobuev@binran.ru (S.V.V.); nadyapsu@binran.ru (N.V.P.); 2 Chemical Analysis and Materials Research Centre, Saint-Petersburg State University, 198504 Saint-Petersburg, Russia; a.misharev@spbu.ru (A.D.M.); e.serebryakov@spbu.ru (E.B.S.)

**Keywords:** fungi, lipids, mass-spectrometry, molecular species

## Abstract

Phosphatidylcholines (PC) are the main membrane lipid constituents comprising more than 50% of total glycerophospholipids. They coordinate a number of cell functions, particularly cell growth, homeostasis, secretion, recognition and communication. In basidial fungi PC are synthesized via the Kennedy pathway as well as through methylation of phosphatidylethanolamines (PE) and then undergo remodeling in Lands cycle that replaces fatty acids in PC molecules. The molecular profile of PC is determined by the genetic features that are characteristic for every species and depend on the environment. Here we present the results of ESI-MS based analyses of PC profiles of 38 species of basidiomycetes belonging to Agaricales (12), Polyporales (17), Russulales (5), Gleophyllales (2), Cantharellales (1), Auriculariales (1), Phallales (1). Although the variety of PC molecular species of basidiomycetes is rather diverse (20–38 molecular species in every profile), only 1–3 main molecular species represent 70–90% of total PC content. The most abundant of them are C36:4 and C36:3, followed by C34:1, C34:2, C36:5, C36:2. In the majority of basidiomycetes, C36:4 reaches up to 50–70% of total PC molecular species. Based on the results of hierarchical cluster analysis four main types of PC profiles which characterized the studied fungi independently from their taxonomic position, ecology, trophic status, and hyphal differentiation have been revealed. Comparative analyses of studied fungi using PCA method have shown that species of Polyporales differ from those of Agaricales by higher variability of PC profiles.

## 1. Introduction

Glycerophospholipids (GPL) are essential structural components of cellular and subcellular membranes acting as an effective permeability barrier. They determine the physical properties of membranes and membrane associated processes like trafficking, fusion and fission [1]. In addition, GPL are second messengers in signal transduction, and effectors of protein structure and function. Particularly they operate as chaperones in protein folding and modulators of transport proteins, receptors, ion channels and enzymes [2,3]. Enormous structural diversity of GPL arises from the combinations of the two fatty acids (vary in lengths mostly between 16–24 carbons and degree of unsaturation), the linkage at the sn-1 position of the glycerol backbone (acyl, alkyl or alkenyl) and the polar head group [4]. Depending on the head group, GPL are differentiated into several classes, where phosphatidylcholines (PC) and phosphatidylethanolamines (PE) are the major GPL, whereas phosphatidylinositols, phosphatidylserines, phosphatidylglycerols, and phosphatidic acids are the minor ones. GPL are unevenly distributed within membranes and may be organized into micro- and nano-domains [5]. PC associate with outer-leaflet of membranes to a greater extent than PE and minor GPL which more often relate to inner-leaflet [6].

In basidial fungi, like in the majority of eukaryotic organisms, PC and PE are the most abundant phospholipid classes, representing 60–80% of the total GPL [7,8,9]. The relative content of PC and PE changes in ontogenesis, as well as under the influence of various biotic and abiotic factors. In some species of basidiomycetes in certain periods of development or adaptation to changing environmental and metabolic conditions PC become the predominant class of GPL (2–4 times higher than the content of PE). For example, PC oversynthesis was recorded at the beginning of the development of the surface culture of *Flammulina velutipes*, characterized by a large number of actively growing undifferentiated hyphae [10]. Similarly in ascomycete *Aspergillus nidulans* direct linear correlations between PC content and hyphal extension rate and branching were observed [11]. In cell cultures and animal models an essential role of PC in cell cycle progression was shown. The activity of PC synthesis increased as cells go through G0/G1 transition. Alternatively, the reduction of PC synthesis was a common feature of apoptosis that could be partially reversed by supplementing cells with PC [12]. An increase in PC concentration has been repeatedly shown under stress conditions. The PC level increased under the influence of heat shock and freezing-thawing of the mycelium of domestic fungus *Serpula lacrymans* [13]. Even during phosphate starvation, when the processes of GPL biosynthesis are sharply reduced, PC accumulation may continue or intensify [7]. Change in the ratio of PC and PE is an effective way to regulate membrane fluidity. This molecular mechanism is based on differences in the form of these lipids, i.e., cylinder shaped PC pack more easily than conical PE which form packing gaps [14].

PC relate with the cell secretion, recognition and communication [15] (Furse, de Kroon, 2015). Mostly they are essential for ER secretion and membrane expansion as well as extracellular vesicles production [16]. Since extracellular vesicles are surrounded by a lipid membrane, changes in the content of GPL, including the elevated PC level, modulate their size and biological activity. In *Candida albicans* genes involved in de novo phospholipid synthesis affected secretion through the changes in both cell wall integrity and release of extracellular vesicles [17]. PC level may be crucial for symbiosis with the host plants because participation into cell to cell interactions [18].

**Figure 1:**
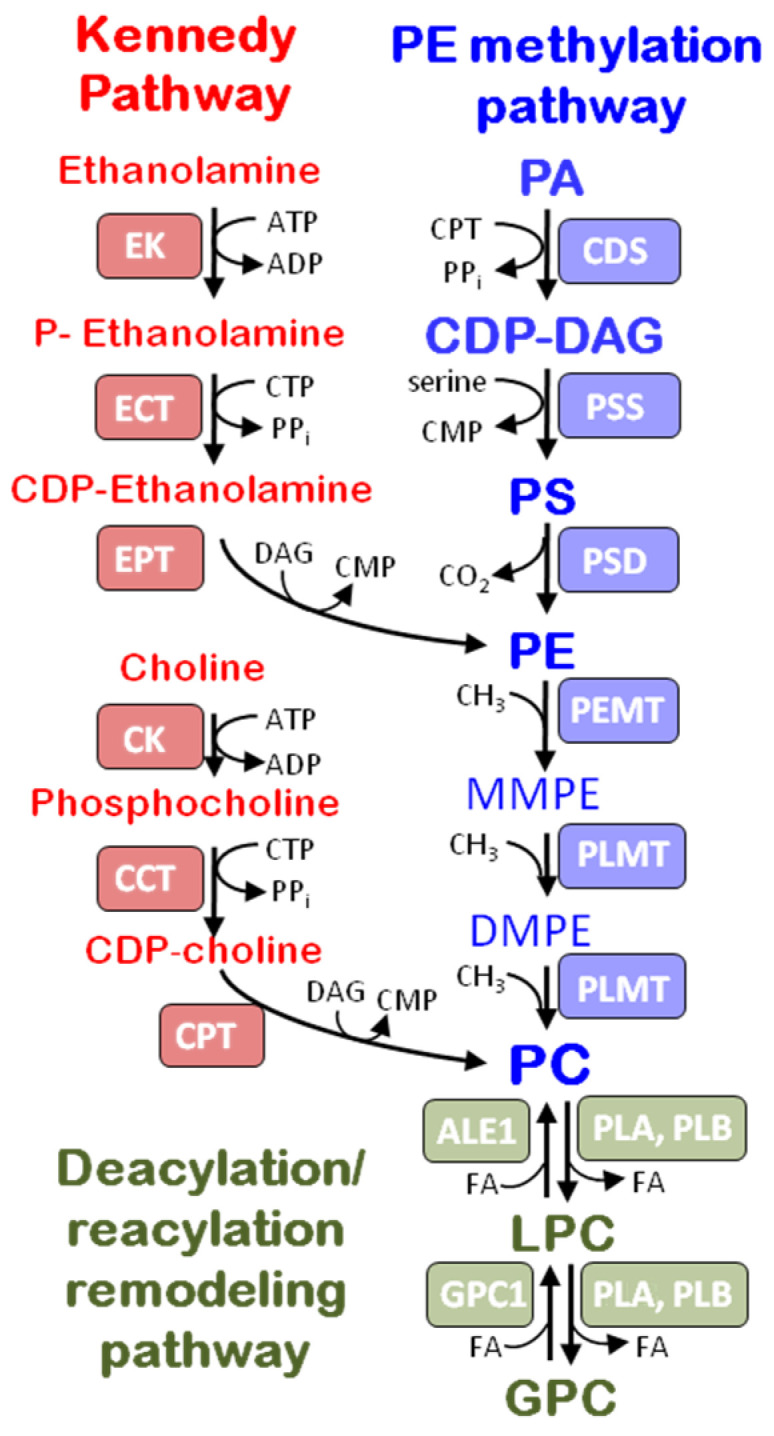
PC metabolism in basidiomycete fungi. The scheme is designed on the basis of published data on the genes and proteins from basidial fungi (Yamashita et al., 2014; Senik et al., 2015; Glab et al., 2016) as well as databases (GenBank NCBI). DAG, diacylglycerol; DMPE, Dimethylethanolamine; GPC, glycerophosphocholine; LPC, lysophosphatidylcholine; MMPE, Monomethylethanolamine; PA, phosphatidic acis; PC, phosphatidylcholine; PS, phosphatidylserine. The Kennedy pathway enzymes (EK, ethanolamine kinase; ECT, phosphoethanolamine cytidylyltransferase; EPT, ethanolaminephosphotransferase; CK, choline kinase; CCT, phosphocholine cytidylyltransferase; CPT, cholinephosphotransferase). The CDP-DG pathway enzymes (PSS, PS synthase; PSD, PS decarboxylase; PEMT, PE methyltransferase; and PLMT, phospholipid methyltransferase). Deacylation/reacylation remodeling pathway enzymes (PLB, phospholipase B; ALE1, lysophospholipid acyltransferase; GPC1, GPC-acyltransferase.

PC synthesis via the CDP-choline pathway (the Kennedy pathway), which consists of three enzyme reactions that convert free choline to PC, have been extensively studied (Figure 1). They include phosphorylation of choline, transfer of CMP to phosphocholine with the formation of CDP-choline, and transfer of phosphocholine from CDP-choline to diacylglycerol [16]. The steps of PC biosynthesis associated with PE N-methyltransferase activity consist of successive methylation of the amino head group of PE (PE methylation pathway). In contrast to the yeasts, in the cells of filamentous basidiomycetes this pathway seems to be apparently inactive. For example, in *Flammulina velutipes*, the ratio of *CHO2* (encode first N-methyltransferase) expression was 10 times less than that of *CPT1* (encode choline phosphotransferase) [7]. PC produced through Kennedy and PE methylation pathways are feature modified via the Lands cycle with participation of phospholipases and lysophospholipid acyl transferases. The substrate specificity of the acyl transferases was admitted to be one of the determining factors of the diversity of PC. It was also assumed that different pathways may synthesize different pools of PC molecular species [19,20].

The PC profiling of filamentous basidiomycetes is barely performed in contrast to other groups of organisms that are characterized by different methods of lipidomic analysis [21]. As far as we are aware, only few reports on the PC structural diversity of basidial fungi have been presented to date. In phytopathogenic basidial fungi from genus *Puccinia* the major PC species were 34:2 (16:0/18:2 (in the author’s edition, the data are presented as 16:0_18:2 and so on [9])) and 36:4 (16:0/20:4) for *P. malvacearum* and 32:0 (16:0/16:0), 34:1 (16:0/18:1), 34:2 (16:0/18:2), 36:2 (18:0/18:2) for *P. glechomatis* [9]. Such PA profiles with a high level of 34 carbon containing molecular species are quite typical for yeast, but seem to differ from filamentous basidiomycetes with a high proportion of C18:2, C18:1, the combination of which usually leads to the biosynthesis of C36:3 and C36:4 molecular species. To what extent this distribution (with high level of 34 carbon containing species) represented in other basidiomycetes species remains unclear. Therefore, the aim of this work was to determine the extent of structural diversity of PC among the species diversity of basidiomycetes with a focus on Agaricomycetes. These fungi possess of wide spectrum of biological active compounds, many of them are edible or medicinal and they are intensively cultivated all over the world [22]. We desired to type the PC profiles in search of their regularities and reasons that constitute a certain PC pattern.

## 2. Materials and Methods

### 2.1. Fungal Strains and Culture Conditions

In this study we used 39 dikaryon strains of 38 species from the Basidiomycetes culture collection of the Komarov Botanical Institute of the Russian Academy of Sciences (LE-BIN) (http://ccinfo.wdcm.org/index.php/collection/by_id/1015/, accessed on 30 December 2021). Taxonomical verification of the strains was done using DNA sequence analysis of the nuclear ribosomal internal transcribed spacer (nrITS) region as previously described [8]. Generated sequences were deposited in GenBank NCBI (https://www.ncbi.nlm.nih.gov/, accessed on 30 December 2021). Their NCBI accession numbers are presented in the Table S1.

The data on geographic origin and substrate of strains are listed in the Table S1. Trophic groups and hyphal systems of species were defined by analyzing of literature and personal observations of the authors.

All strains were grown on beer wort agar (4% beer wort, Severnaya Pivovarnya, Russia; 2% agar Difco, USA) at 25 °C in the dark until mycelia reached the edge of the Petri dishes.

### 2.2. Lipid Extraction and Fractionating

The fresh fungal mycelium was scrapped from the Petri dish with a scalpel and homogenized in isopropanol. Total lipids were extracted according to the Nichols method [23] with modifications as previously described [8]. Briefly, the homogenized mycelium was kept in isopropanol at 70 °C for 30 min, and then extracted a second time with isopropanol-chloroform (1:1). The combined extracts were evaporated, dissolved in chloroform–methanol (1:1), and washed clean of water-soluble impurities with a 2.5% NaCl solution. The obtained extracts were evaporated, redissolved in a mixture of chloroform–methanol (1:1) and stored at −20 °C until analysis.

PC were separated by two dimensional thin-layer chromatography (TLC) on silica gel 60 10 × 10 cm plates (Merck, Darmstadtcity, Germany) in a solvent system of chloroform–methanol–water (65:25:4) in the first direction and chloroform–acetone–methanol–acetic acid–water (50:20:10:10:5) in the second direction [24]. After temporary visualization in iodine vapors PC spots were scrapped from TLC plates and eluted with chloroform–methanol (1:1) at 4 °C overnight, then evaporated and redissolved in methanol.

### 2.3. ESI-MS Analyses of PC Molecular Species

The molecular PC species were determined by high-resolution accurate mass MS using the micrOTOF TOF mass spectrometer (Bruker Daltonics GmbH, Bremen, Germany) equipped with an electrospray ionization (ESI) source, the capillary voltage of the ion source was set at 4000 V, the nebulizer gas pressure was 0.4 bar, and the drying gas flow was 4.0 L/min with temperature of 180 °C. The spectra were recorded in the positive ion mode during continuous direct probe injection in the range of 150–1000 *m*/*z* within 0.5 s scan duration. Sodium formate cluster ions were used to calibrate *m*/*z* scale before every analysis. The height of detected monoisotopic peak was used for quantification after isotope correction. ESI-MS spectrum data were processed using Compass Data Analysis Viewer 4.4. software (Bruker Daltonics) for identification and quantification with the external standard mixture of PC 13:0/13:0, PC 16:0/16:0, PC 18:1/18:2, PC 18:2/18:2 (LarodanFine Chemicals, Malmo, Sweden). Peak annotation was carried out using LipidMaps [25]. False positives are checked manually. A mass tolerance of 5 ppm was used to identify the lipid species, which were annotated as sum of carbon atoms in the fatty acids: sum of double bonds in the fatty acids (e.g., 36:4). For quantification ESI-MS data were normalized relative to the total heights expressed in the intensities (arbitrary units), and the percentage of each molecular species was calculated as follows:

$$\%=\frac{A(peak\left(m/z\right))}{\sum A\left(peaks\right)}\;\times \;100,$$

where *A* (*peak*) denotes to the intensity (arbitrary units) of each identified peak and ∑A(peaks) refers to the sum of the intensities of each PC peak. To correct analytical data, the external standard mixture was injected regularly after each ten samples. According to the intensity values of its components the sensitivity factor (F) taken as F = 1 for every PC molecular species.

### 2.4. Statistics

Statistical analyses was processed in the environment of the R language 4.1.0 [26]. Contents of PC molecular species were normalized per sample sum (sum = 100). For exploratory analysis of 39 fungal strains belonging to 38 species Principal Component Analysis (PCA) was performed with «pcaMethods» [27]. For this, unit variance scaling (SD = 1) and mean centering (M = 1) were made. SVD algorithm was used. To test difference between orders in a new space, PERMANOVA test with the Euclidean distances in the «vegan» package [28] was performed. For 6 predominant PC species heatmap combined with hierarchical clustering (HCA) was made with «ComplexHeatmap» package [29]. For clustering Ward algorithm and Euclidean distances were used. Data were unit variance scaled and mean centered.

## 3. Results and Discussion

### 3.1. PC Molecular Species Diversity

We have demonstrated the distribution of various PC molecular species in the members of Agaricomycetes class. Studied strains belong to 38 species, 26 families and 6 orders according to systema He et al. (2019) [30] (Table 1). The most strains split between two largest orders Agaricales (12 strains) and Polyporales (17 strains), whereas the rest orders count from 1 (Auriculares, Cantharellales, Phallales) to 5 (Russulales) strains. The majority of strains (34) relate to saprothroph fungi (S) and 5 strains—to biotroph ones (P), which are parasites on trees (Le) and mushrooms (Mm). Among saprothrophs, the xylotrophic fungi (Le) are dominated, 5 species inhabit on leaf-litter (St), 3—on humus and 1 species was collected on equine dung. In total, 29 species colonize different wooden substrates from living tree to very rotten fallen trunks including such specific substrates as pine cones and bark. Xylotrophic fungi are divided on species causing white (WR) or brown (BR) rot of wood according their enzymatic complexes and the ways of wood destroying. These features of strains are listed in the Table S1.

PC profiles of each fungal strain included 14–27 molecular species with the degree of unsaturation ranging from 0 to 7 (Table S1). The 36:4, 36:3, 36:2, 34:3, and 34:2 were the most widespread molecular species, detected in almost all strains (Table 1). Less abundant species found in some fungal strains included 34:1, 36:5, 33:2, and 35:3. The rarest molecular species of PC were 32:2, 34:4, 35:4, 35:2, 36:6, 38:2, and some oxygenated and unidentified species.

The molecular profiles of PC like other phospholipids are limited by the genetic features that are characteristic for every species and genus and depend on the environmental conditions [9]. For PC of ascomycetous yeasts, particularly for well-documented *Saccharomyces cerevisiae*, 32:2 and 34:2 are the most abundant, followed by 32:1 and 34:1 [1,21,31,32,33]. In ascomycetous pathogenic fungus *Metarhizium robertsii* in addition to the rather high level of 34:1, 34:2, 34:3 many 36 carbon containing (36:x) PC molecular species have been identified [34]. Recently we have also demonstrated an equal distribution of 34:x and 36:x for other ascomycetous pathogen *Stagonospora cirsii* [35]. But in filamentous ascomycetes such as *Trichoderma reesei*, *Aspergillus nidulans* and *Neurospora crassa* the content of 36:4, particularly 18:2/18:2, was 60–75% of the total PC [36]. Among other dominated PC species 5–15% 36:3 (18:2/18:1) and 5–20% 34:2 (16:0/18:2) were revealed. Mentioned above distribution of molecular species is very similar to that observed in the basidiomycetes studied here. The reasons for this similarity are likely to be some aspects of cell morphology and physiology, as well as enzymatic activity. Indeed, the membrane fluidity, packing density and permeability may affects the functioning of membrane-associated biomolecules, including enzymes. PC of different structure, particularly 34:x and 36:x can have considerably different action on diverse membrane-associated processes.

Very little is still known about diversity of PC molecular species in basidiomycetes. In teliospores of two species from genus *Puccinia* (class Pucciniomycetes) 16:0/18:2 and/or 16:0/18:1 were the main molecules accompanied by 16:0/20:4, 16:1/20:4 and some other species bearing unsaturated and very long chain fatty acids [9]. In six very specific pathogenic species *Malassezia* (class Ustilagomycetes), which characterized by the absence of de novo synthesis of fatty acids, PC molecular species were distributed as follows 36:2 > 36:3 >> 36:4, 34:1, 34:2 [37]. According to our results in all 38 species of investigated basidiomycetes 36:x is an only dominant group of molecular species of PC, while 34:x, 38:x and 40:x are minor ones.

Compared to many higher eukaryotes, the fatty acid composition of fungi, including yeasts and filamentous basidiomycetes, is not very diverse. This results in few combinations of acyl groups in PC molecules. For example, in yeast *Saccharomyces cerevisiae* PC 34:1 has been revealed to consist of C16:0 and C18:1 (predominant acyl chain combination) or C18:0 and C16:1. PC 34:2 contains C16:1 and C18:1 fatty acid residues [33]. A number of studies reported that homeostatic control of the PC molecular composition is regulated by cross-talk between biosynthetic pathways and remodeling [14,38]. Particularly, a high level of PC 16:0/18:1 in yeast is associated with postsynthetic remodeling events. It was shown that the activity of glycerophosphocholine acyltransferase (Gpc1) is responsible for the replacement of 32:2 and 34:2 by 32:1 and 34:1. This enzyme preferably affects PC profile. Loss of Gpc1 decreased the level of PC 32:1 and PC 34:1 and increased those of PC 32:2 and PC 34:2, while other GPL were unchanged [33]. In contrast to lysophospholipid acyltransferase Ale1, which catalyzes the addition of preferably unsaturated fatty acid at the sn-2 position of Lyso-PC, Gpc1 catalyzes the addition of fatty acid (saturated or monounsaturated) to the sn-1 position of glycerophosphocholine. Particularly, with the assistance of Gpc1 the remodeling with the replacement of 36:4 by 34:2 and 36:2, 36:3 becomes possible. There is no information about distribution of Gpc1 among species of Basidiomycota. At the same time, GPCAT homologies were found in the major eukaryotic organisms including species of Ascomycota [39]. Our data about species with high proportion of 34:2 (possibly 16:0/18:2) such as Steccherinum ochraceum and 36:3 (possibly 18:1/18:2) such as *Sparassis crispa* and *Tyromyces lacteus*, that is untypical for the majority of basidiomycetes, may be of interest to confirm a role for Gpc1 in PC biosynthesis in basidial fungi.

### 3.2. Comparative Analyses, Typing and Clustering of PC Profiles

**Figure 2:**
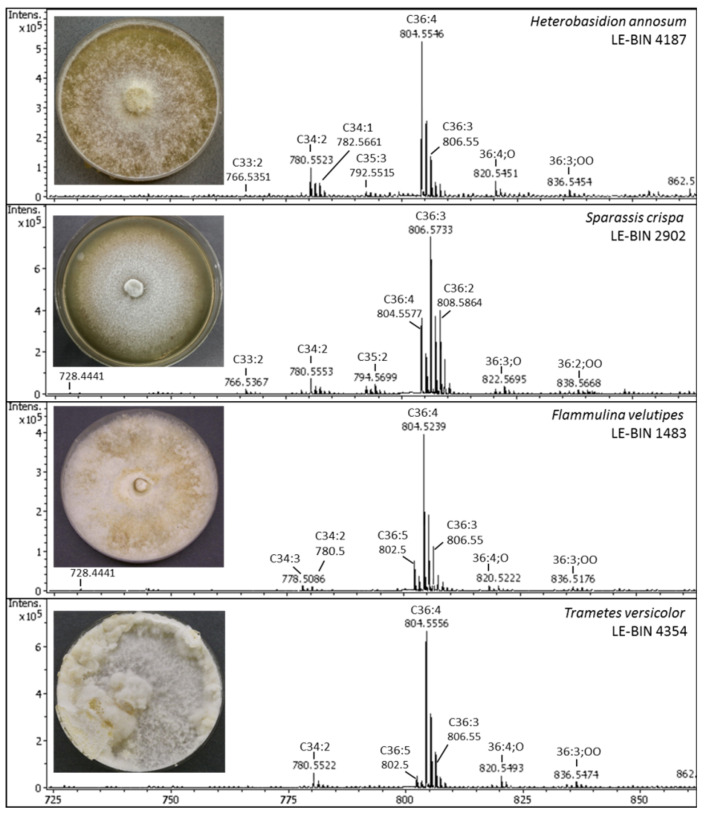
ESI-MS mass-spectra of phosphatidylcholine profiles of basidial fungi.

The main differences in PC profiles in studied strains included the diverse ratio of 36:4 to 36:3 PC, and the various relative abundance of 36:5 PC and C34:X PC. Figure 2 depicts the mass-spectra of contrast PC profiles. Mass spectrum from *Heterobasidion annosum* demonstrates the elevated level of 34:2 PC. Spectrum from *Sparassis crispa* is interesting because of high level of 36:3 PC, spectrum from *Flammulina velutipes* illustrates high level of 36:5 PC. The mass spectrum from *Trametes versicolor* 4354 is an example of spectra enriched in 36:4 PC.

By the relative amount, 36:4 PC (most likely 18:2/18:2 PC) was the predominant component in the most of fungal species (up to 80% of total PC), followed by 36:3 PC (1–40%), and 36:2 PC (1–23%). Such monodominant PC profiles with 50–80% of 36:4 PC are in accordance with our previous reports and literature data for fatty acid composition in phospholipids of basidiomycetes fungi: C18:2 fatty acid was the only predominant fatty acid in PC of almost all studied fungi, C18:1 does not exceed 20% of total FA [8,40,41,42]. However, the studies conducted on a large number of basidiomycetes where the total fatty acid composition was analyzed showed that in addition to C18:2 many species are characterized by a predominance of C18:1 fatty acids [43,44]. The ratio of these fatty acids varies widely and does not have a clear taxonomic correlation. This parameter, apparently, is not genetically determined, but depends on the environmental conditions. On the contrary, an extremely low content of C18:3 is typical for all basidiomycetes, which is confirmed by numerous literature data [43,44,45]. So the species accumulating relatively high amounts of PC 36:5, PC 36:6, including *Flammulina velutipes* and *Auriscalpium vulgare*, which probably include C18:3, are quite a rare finding.

**Figure 3:**
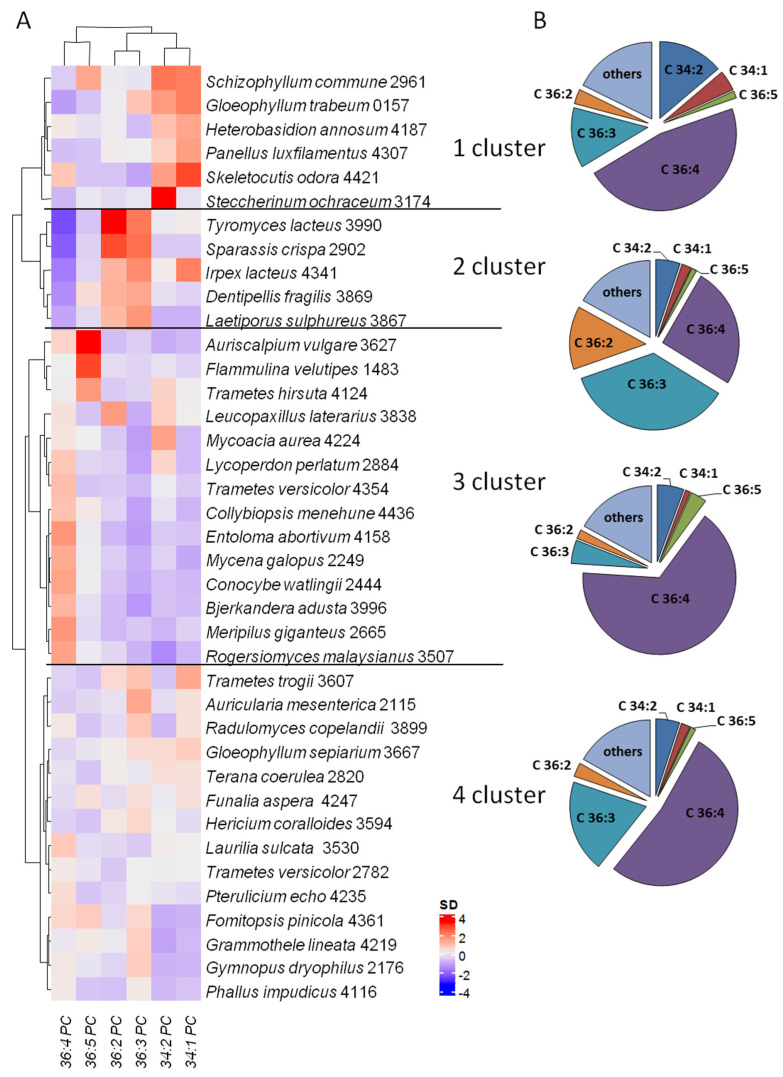
(**A**) Heatmap representing the content of 6 predominant molecular species of phosphatidylcholines. Data are unit varianse scaled (SD = 1) and mean centered (M = 0). The map is combined with a dendrogram of hierarchical clustering performed by Ward’s method in the Euclidean space. Colors represent different concentrations indicated by the color bar. (**B**) Diagrams with mean content of PC molecular species in strains of each cluster.

To reveal PC profiles typical for different fungal groups the hierachical clastering analysis (HCA) was carried out (Figure 3). HCA provides intuitive visualization of a data table. Each colored cell on the map corresponded to autoscaled (SD = 1) and mean centered (M = 0) concentration value in the data table. HCA based on the concentration (%) of six dominant PC molecular species in 39 basidiomycete strains and reveal several clusters.

Cluster 1 contained 6 xylotrophic species from different orders (Polyporales, Agaricales, Russulales, and Gloeophyllales), trophic groups and ecological and phisoplogical traits. A distinctive feature of the species in cluster 1 is higher level of C34:x PC, including C34:2 PC (10–20%). Cluster 2 comprised 5 xylotrophic species, among them 4 strains from Polyporales and 1—from Russulales. The common feature of these fungi is that their hyphal sistems are mono- or dimitic, but not trimitic. A distinctive feature of this cluster is accumulation of 36:3 PC (*m*/*z* 806.5) in the amount of more that 30% from total PC. *Tyromyces lacteus* LE-BIN 3990 and *Sparassis crispa* LE-BIN 2902 were most enriched in 36:3 PC. Cluster 3 included fungi with the highest content of C36:4 PC: 5 species from Polyporales order, 7—Agaricales, 1—Russulales, and 1—Cantharellales. Separate sub-cluster comprises 3 strains—*Flammulina velutipes* LE-BIN 1483, *Auriscalpium vulgare* LE-BIN 3627 and *Trametes hirsuta* LE-BIN 4124 with high level (7–15%) of 36:5 PC (presumedly 18:3/18:2 PC). Cluster 4 consisted of fungal species that had no clear PC profile characteristics including 6 species from Polyporales order, 4—from Agaricales, 2—Russulales, 1—Phallales, and 1—Gloeophyllales.

The cluster analysis indicated that the PC profiles of six fungal species accumulating 34:X molecular species (16:0/18:1, 16:0/18:2) (cluster 1) were closest to five species accumulating 36:3 (18:1/18:2) and 36:2 (18:1/18:1, 18:0/18:2) (cluster 2). The species including *S. commune*, *G. trabeum*, *H. annosum*, *P. luxfilamentus*, *S. odora*, *S. ochraceum*, *T. lacteus*, *S. crispa*, *I. lacteus*, *D. fragilis*, *L. sulphureus* appear to have significant higher individuality than other species. So 34:X, 36:3 and 36:2 can be considered to be the most responsible for the discrimination among clusters. Thus, the results demonstrated that the PC profiles of fungi from Cluster 1 and Cluster 2 had key difference among 39 strains studied.

While the statistical analysis did not revealed vast differences between taxonomical, trophic and ecological groups as well as between the strains that contrast in their growth and morphological features, we did note a few tendencies that may demarcate species with a certain PC profile. It is probable, the most significant differences in PC profiles are noticeable at the higher taxon level (class and above).

**Figure 4:**
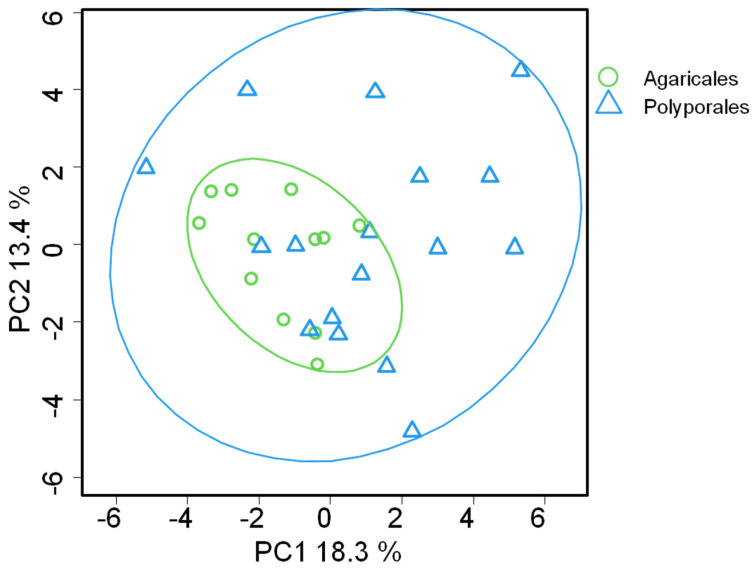
PCA plot analysis of phosphatidylcholine composition in basidiomycete species. Ellipses—95% confidence intervals.

Discriminatory analysis of PC profiles from various basidiomycete strains was analyzed by PCA (Figure 4). Statistical difference between two orders is supported by PERMANOVA test P = 0.02 for first two principal component. The first and second principal components accounted for 13.4% and 18.3% of total sample variance, respectively. This method allowed to discriminate samples according to the taxonomical position of strains. The data represented in Figure 3 demonstrate that variability of PC profiles of Polyporales species exceeds that of Agaricales.

One of the central questions in lipid biology is to understand how existing phospholipid molecules to be the membrane constituents contribute to cell functions. This study adds new details on the structural diversity of PC that form a large part of membranes and its possible role in processes connected with fungal biology such as ecological plasticity and survival. The higher diversity of PC profiles in Polyporales may be due to the fact that species of Polyporales are evolutionary adapted to a wider range of carbon sources (lignin and cellulose) and state of the substrate (live, dead wood). Trophic and ecological plasticity is biochemically supported at many levels, including the organization and composition of cell membranes. The wider variability of PC molecular species is one of the factors that ensure the adaptation of organisms to different environmental conditions, food sources, etc.

## Figures and Tables

**Table 1:** The major molecular species of phosphatidylcholines in basidiomycetes (relative abundance, %).

Order	Family	Species	Trophic Group ^c^	Ecology ^d^	Type of Rot ^e^	Growth ^f^	Hyphal System ^g^	*m*/*z* [M+Na]^+^/Structure ^a^						
								778.5/ 34:3	780.6/ 34:2	782.6/ 34:1	802.5/ 36:5	804.6/ 36:4	806.6/ 36:3	808.6/ 36:2
Agaricales	Entolomataceae	*Entoloma abortivum* 4158	Mm	P	WR	S	MM	0.49	4.33	1.08	1.72	77.83	1.23	0.09
	Lycoperdaceae	*Lycoperdon perlatum* 2884	Hu	S	WR	S	MM	0.65	8.79	0.86	0.84	64.68	2.18	1.76
	Schizophyllaceae	*Schizophyllum commune* 2961	Le	S	WR	F	MM	nd ^b^	15.89	5.00	6.11	45.19	13.48	3.54
	Mycenaceae	*Mycena galopus* 2249	St	S	WR	S	MM	2.09	4.74	0.42	1.83	71.86	4.00	1.54
	Tricholomataceae	*Leucopaxillus laterarius* 3838	Hu	S	WR	M	MM	nd	9.14	2.07	nd	57.04	4.18	11.08
	Bolbitiaceae	*Conocybe watlingii* 2444	Hu	S	WR	S	MM	0.91	3.64	0.79	1.85	74.34	3.20	0.95
	Omphalotaceae	*Gymnopus dryophilus* 2176	St	S	WR	S	MM	1.94	2.82	0.74	1.50	55.64	22.33	2.10
	Omphalotaceae	*Panellus luxfilamentus* 4307	Le	S	WR	S	MM	nd	9.06	4.20	nd	42.01	15.27	4.20
	Omphalotaceae	*Collybiopsis menehune* 4436	St	S	WR	S	MM	6.45	5.71	0.74	2.42	65.84	2.06	1.88
	Pterulaceae	*Radulomyces copelandii* 3899	Le	S	WR		MM	1.81	3.05	2.46	nd	55.58	23.66	2.53
	Pterulaceae	*Pterulicium echo* 4235	Le	S	WR	F	DM	2.00	5.85	1.57	nd	58.30	15.00	2.08
	Physalacriacerae	*Flammulina velutipes* 1483	Le	S	WR	F	MM	1.88	5.77	1.39	10.80	52.79	10.47	2.62
Auriculariales	Auriculariaceae	*Auricularia mesenterica* 2115	Le	S	WR	M	MM	0.92	5.42	2.44	0.93	44.79	30.31	2.97
Cantharellales	Hydnaceae	*Rogersiomyces malaysianus* 3507	St	S	BR	S	MM	0.14	nd	0.78	1.77	74.45	5.17	2.32
Gloeophyllales	Gloeophyllaceae	*Gloeophyllum trabeum* 0157	Le	S	BR	M	DM	1.86	13.34	5.02	nd	33.94	24.19	3.66
	Gloeophyllaceae	*Gloeophyllum sepiarium* 3667	Le	S	BR	M	TM	1.63	8.23	2.99	1.48	47.09	19.57	4.25
Phallales	Phallaceae	*Phallus impudicus* 4116	Hu	S	WR	S	MM	1.27	3.15	1.06	nd	55.07	16.73	0.72
Polyporales	Laetiporaceae	*Laetiporus sulphureus* 3867	Le	P	BR	M	DM	0.23	2.69	0.65	0.98	35.90	33.74	8.66
	Irpecaceae	*Irpex lacteus* 4341	Le	S	WR	F	DM	2.69	7.06	4.95	0.76	26.26	35.59	8.98
	Dacryobolaceae	*Tyromyces lacteus* 3990	Le	S	BR	S	MM	3.48	6.19	2.14	nd	15.44	39.23	22.97
	Polyporaceae	*Grammothele lineata* 4219	Le	S	WR	F	DM	1.10	1.66	0.83	2.44	50.60	21.89	3.47
	Polyporaceae	*Trametes versicolor* 2782	Le	S	WR	F	TM	1.37	6.43	2.01	1.41	54.88	15.33	1.21
	Polyporaceae	*Trametes versicolor* 4354	Le	S	WR	F	TM	1.27	6.34	1.21	nd	67.04	6.39	1.34
	Polyporaceae	*Trametes hirsuta* 4124	Le	S	WR	M	TM	2.03	8.94	1.96	6.94	52.12	10.64	1.39
	Polyporaceae	*Trametes trogii* 3607	Le	S	WR	F	TM	3.75	3.89	3.93	nd	46.52	24.55	5.81
	Polyporaceae	*Funalia aspera* 4247	Le	S	WR	M	TM	1.34	6.17	2.43	2.94	48.45	18.65	2.60
	Phanerochaetaceae	*Terana coerulea* 2820	Le	S	WR	M	MM	0.64	8.09	2.44	nd	49.72	13.89	4.27
	Phanerochaetaceae	*Bjerkandera adusta* 3996	Le	S	WR	F	MM	0.75	3.75	0.88	1.26	69.20	0.18	0.29
	Steccherinaceae	*Steccherinum ochraceum* 3174	Le	S	WR	S	DM	2.31	21.52	1.70	1.62	40.05	13.84	2.52
	Fomitopsidaceae	*Fomitopsis pinicola* 4361	Le	S	BR	F	DM	nd	2.39	0.67	4.05	59.82	20.20	2.43
	Sparassidaceae	*Sparassis crispa* 2902	Le	P	BR	S	MM	0.96	4.32	1.20	0.58	19.21	40.69	16.69
	Meruliaceae	*Mycoacia aurea* 4224	Le	S	WR	M	MM	0.84	12.74	0.86	2.00	56.14	1.45	1.19
	Meripilaceae	*Meripilus giganteus* 2665	Le	P	WR	F	MM	nd	2.84	1.34	0.96	77.65	8.43	0.20
	Incrustoporiaceae	*Skeletocutis odora* 4421	Le	S	WR	S	DM	nd	12.86	6.18	nd	64.69	2.47	0.93
Russulales	Auriscalpiaceae	*Auriscalpium vulgare* 3627	St	S	WR	S	DM	1.31	2.35	0.81	15.42	60.71	9.84	0.48
	Hericiaceae	*Hericium coralloides* 3594	Le	S	WR	S	MM	1.49	6.68	1.55	nd	46.05	20.44	4.70
	Hericiaceae	*Dentipellis fragilis* 3869	Le	S	WR	M	MM	2.53	5.61	1.37	3.11	30.34	30.30	9.31
	Bondarzewiaceae	*Heterobasidion annosum* 4187	Le	P	WR	M	DM	1.62	10.33	4.01	1.29	54.60	6.91	3.68
	Bondarzewiaceae	*Laurilia sulcata* 3530	Le	S	WR	M	TM	0.71	7.06	2.06	1.08	63.89	9.27	2.32

Notes. Phosphatidylcholine molecular species were analyzed using ESI-Q-TOF-MS performed with a micrOTOF (Bruker Daltonics) mass spectrometer after separation of the lipid classes by TLC as described in Materials and methods. Major molecular species are detailed here, with further information on minor species listed in Supplementary Table S2. ^a^ Molecular structures provide total carbon number, number of double bonds, such as 36:4. ^b^ nd means not detected. ^c^ Trophic groups: Mm—parasite on mushrooms, Le—Xylotrophic, St—Litter-Saprotrophic, Hu—Humus-Saprotrophic. ^d^ Ecology: S—saprotroph, P—parasite. ^e^ Type of Rot: WR—white rot, BR—brown rot. ^f^ Growth: F—fast (9 cm Petri dish—1 week), M—medium (9 cm Petri dish—2–3 week), S—slow (9 cm Petri dish—4 week or more) ^g^ Hyphal system: MM—monomitic, DM—dimitic, TM—trimitic.

## Supplementary Materials

The following supporting information can be downloaded at: https://www.mdpi.com/article/10.3390/jof8020177/s1, Table S1: Annotated list of studied strains, Table S2: Composition of phosphatidylcholine molecular species.
