# Supplementary material for: Diversity of ESI-MS Based Phosphatidylcholine Profiles in Basidiomycetes
Source: J Fungi (Basel). 2022 Feb 11;8(2):177. doi: 10.3390/jof8020177 (PMC8879007; doi:10.3390/jof8020177)
Supplement: Supplementary file 1 [file jof-08-00177-s001.zip › Table S1.pdf]

Table S1. Annotated list of the studied strains.

Order	Family	Species, strain number	GenBank accession number	Isolation date	Geographical origin	Substrate
Agaricales	*Bolbitiaceae*	*Conocybe watlingii* Hauskn., LE-BIN 2444	OM044593	20-Aug-2009	Russia, Krasnodar Territory, Caucasus Biosphere Reserve, 43°53'27.7"N, 040°39'45.8"E	equine dung
Agaricales	*Entolomataceae*	*Entoloma abortivum* (Berk. & M.A. Curtis) Donk, LE-BIN 4158	OM057664	22-Aug-2018	Russia, Tomsk Region, 56°45'36.1"N, 084°43'27.8"E	
Agaricales	*Lycoperdaceae*	*Lycoperdon perlatum* Pers., LE-BIN 2884	OM044595	13-Aug-2012	Russia, Karachayevo-Circassian Republic, in vic. of Dombay settlement, 43°17'02.2"N, 041°37'08.6"E	on litter
Agaricales	*Mycenaceae*	*Mycena galopus* (Pers.) P. Kumm., LE-BIN 2249	MK795846	07-Oct-2007	Russia, Leningrad Region, Vyborg District, Gor'kovskaya settlement	in mosses
Agaricales	*Mycenaceae*	*Panellus luxfilamentus* A.L.C. Chew & Desjardin, LE-BIN 4307	OM057666	12-Nov-2018	Vietnam, Cao Bằng Province, Phia Oắc - Phia Đén National Park, 22°35'38.7"N, 105°53'28.2"E	on fallen branch
Agaricales	*Omphalotaceae*	*Collybiopsis menehune* (Desjardin, Halling & Hemmes) R.H. Petersen, LE-BIN 4436	OM044597	16-May-2019	Vietnam, Đắk Lắk Province, Chư Yang Sin National Park, 12°23'47.726"N, 108°20'56.960"E	on soil
Agaricales	*Omphalotaceae*	*Gymnopus dryophilus* (Bull.) Murrill, LE-BIN 2176		04-Sep-2006	Russia, Krasnodar Territory, Sochi urban District, Caucasus Biosphere Reserve	on beech litter
Agaricales	*Physalacriaceae*	*Flammulina velutipes* (Curtis) Singer, LE-	KM668876	Oct-1982	Russia, Saint Petersburg city, Kalininsky District	on poplar

		BIN 1483				
Agaricales	*Pterulaceae*	*Pterulicium echo* (D.J. McLaughlin & E.G. McLaughlin) Leal-Dutra, Dentinger & G.W. Griff., LE-BIN 4235	OM057665	29-Oct-2018	Vietnam, Bình Phước Province,<br>Bù Gia Mập National Park	
Agaricales	*Pterulaceae*	*Radulomyces copelandii* (Pat.) Hjortstam & Spooner, LE-BIN 3899	MG722738	12-Jul-2016	Georgia, Republic of Abkhazia, Gudauta District, Pitsunda-Myussera Biosphere Reserve, 43°09'43.3"N, 040°27'15.4"E	on fallen branch of oak
Agaricales	*Schizophyllaceae*	*Schizophyllum commune* Fr., LE-BIN 2961	OM044596			
Agaricales	*Tricholomataceae*	*Leucopaxillus laterarius* (Peck) Singer & A.H. Sm., LE-BIN 3838	MG720453	25-Aug-2015	Russia, Krasnoyarsk Territory, Sayano-Shushensky Biosphere Reserve, 52º06′28″N, 092º13′53.5″E	on litter among pines
Auriculariales	*Auriculariaceae*	*Auricularia mesenterica* (Dicks.) Pers., LE-BIN 2115		06-Oct-2006	Russia, Rostov Region, Sholokhovsky District, vic. of Kalininsky settlement, 49°31'36.1"N, 041°50'28.0' E	
Cantharellales	*Hydnaceae*	*Rogersiomyces malaysianus* (K. Matsush. & Matsush.) Zmitr., LE-BIN 3507	KU820988	21-May-2014	Vietnam, Đắk Lắk Province, Chư Yang Sin National Park, 12°24'31"N, 108°23'19"E	on bark of living tree of Dalat pine
Gloeophyllales	*Gloeophyllaceae*	*Gloeophyllum sepiarium* (Wulfen) P. Karst., LE-BIN 3667	KY352494	05-Aug-2015	Russia, Moscow Region, vic. of the Zvenigorod Biological Station of Moscow State University, 55º41′58″N, 036º43′23″E	burned stump of spruce

Gloeophyllales	*Gloeophyllaceae*	*Gloeophyllum trabeum* (Pers.) Murrill, LE-BIN 0157	KY433982	1967	Germany, VKMF-433	
Phallales	*Phallaceae*	*Phallus impudicus* L., LE-BIN 4116		29-Aug-2018	Russia, Bryansk Region, “Bryansk Forest” Nature Reserve, 52º31'31.2"N, 034º04'36.0"E	on litter
Polyporales	*Fomitopsidaceae*	*Fomitopsis pinicola* (Sw.) P. Karst., LE-BIN 4361	OM033739	22-Aug-2019	Russia, Orel Region, Mtsensk District, vic. of Volya settlement	on fallen trunk of apple-tree
Polyporales	*Incrustoporiaceae*	*Skeletocutis odora* (Sacc.) Ginns, LE-BIN 4421	OM065337	19-Aug-2020	Russia, Bryansk Region, Trubchevsk District, “Bryansk Forest” Nature Reserve	on fallen trunk of pine
Polyporales	*Incrustoporiaceae*	*Tyromyces lacteus* (Fr.) Murrill, LE-BIN 3990	OM033736	23-Aug-2017	Russia, Bryansk Region, “Bryansk Forest” Nature Reserve, 52º49′38.1″N, 043º97′34.0″E	on fallen trunk of alder
Polyporales	*Irpicaceae*	*Irpex lacteus* (Fr.) Fr., LE-BIN 4341	OM033738	19-Aug-2019	Russia, Belgorod Region, Korocha District, 50°52′56.6″N, 37°11′27.6″E	on living trunk of cherry-tree
Polyporales	*Irpicaceae*	*Trametes trogii* Berk. LE-BIN 3607	OM065336	11-Aug-2015	Russia, Bryansk Region, “Bryansk Forest” Nature Reserve, 52º26′54″N, 033º51′36″E	on fallen wood of aspen
Polyporales	*Laetiporaceae*	*Laetiporus sulphureus* (Bull.) Murrill, LE-BIN 3867	MG734830	26-Aug-2016	Russia, Republic of Tatarstan, “Privolzhskoe Lesnichestvo” forestry, 55°44′48.2″N, 048°47′26.2″E	on fallen rotten trunk of oak
Polyporales	*Meripilaceae*	*Meripilus giganteus* (Pers.) P. Karst., LE-BIN	OK641916	08-Sep-2010	Russia, Republic of Adygeya, Maikop District, 44°09'26.3"N, 040°08'49.2"E	in soil at large old stump of beech
Polyporales	*Meruliaceae*	*Mycoacia aurea* (Fr.)	OM057669	28-Oct-2018	Vietnam, Bình Phước	on fallen

		J. Erikss. & Ryvarden, LE-BIN 4224			Province, Bù Gia Mập National Park, 12°11'33.6"N, 107°12'38.8"E	branch
Polyporales	*Phanerochaetaceae*	*Bjerkandera adusta* (Willd.) P. Karst., LE-BIN 3996	OM044598	24-Aug-2017	Russia, Bryansk Region, "Bryansk Forest" Nature Reserve, 52º51′44.2″N, 034º07′77.9″E	on fallen trunk of aspen
Polyporales	*Phanerochaetaceae*	*Terana coerulea* (Lam.) Kuntze, LE-BIN 2820	OL840640	01-Sep-2011	Russia, Primorye Territory, Leopard's Land National Park, 43°23'08.15"N, 131°32'13.65"E	on fallen twigs of deciduous tree
Polyporales	*Polyporaceae*	*Funalia aspera* (Jungh.) Zmitr. & Malysheva, LE-BIN 4247	OM057668	30-Oct-2018	Vietnam, Bình Phước Province, Bù Gia Mập National Park, 12°11'24.1"N, 107°11'29"E	on fallen branch
Polyporales	*Polyporaceae*	*Grammothele lineata* Berk. & M.A. Curtis, LE-BIN 4219	OM057667	26-Oct-2018	Vietnam, Bình Phước Province, Bù Gia Mập National Park, 12°11'28.2"N, 107°12'21.7"E	on fallen deciduous branch
Polyporales	*Polyporaceae*	*Trametes hirsuta* (Wulfen) Lloyd, LE-BIN 4124	OM033737			
Polyporales	*Polyporaceae*	*Trametes versicolor* (L.) Lloyd, LE-BIN 2782	OM033552			
Polyporales	*Polyporaceae*	*Trametes versicolor* (L.) Lloyd, LE-BIN 4354	OL819880	21-Aug-2019	Russia, Orel Region, Glazunovka District, 52º30'28.6"N, 036º13'25.9"E	on apple-tree trunk
Polyporales	*Sparassidaceae*	*Sparassis crispa* (Wulfen) Fr., LE-BIN 2902	KJ183188	14-Aug-2012	Russia, Karachayevo-Circassian Republic, Teberda State Nature Biosphere Reserve	on rotten wood (spruce?)

Polyporales	*Steccherinaceae*	*Steccherinum ochraceum* (Pers. ex J.F. Gmel.) Gray, LE-BIN 3174	KY321177	01-Aug-2013	Russia, Kaluga Region, Kaluzhskiye Zaseki State Nature Reserve	on fallen branch of aspen
Russulales	*Auriscalpiaceae*	*Auriscalpium vulgare* Gray, LE-BIN 3627	OM044594	29-Aug-2015	Russia, Krasnoyarsk Territory, Sayano-Shushensky Biosphere Reserve, 52º08′44″N, 092º16′38″E	pine cone
Russulales	*Bondarzewiaceae*	*Heterobasidion annosum* (Fr.) Bref., LE-BIN 4187	OK641917	22-Aug-2018	Russia, Tomsk Region, 56°46'33.4"N, 084°43'31.4"E	trunk of spruce
Russulales	*Bondarzewiaceae*	*Laurilia sulcata* (Burt) Pouzar, LE-BIN 3530	MG722733	27-Jul-2013	Russia, Republic of Buryatia, Barguzinsky Biosphere Reserve, 54º18′04″ N, 109º35′14″ E	large fallen trunk of Siberian pine
Russulales	*Hericiaceae*	*Dentipellis fragilis* (Pers.) Donk, LE-BIN 3869	MG734825	26-Aug-2016	Russia, Republic of Tatarstan, “Privolzhskoe Lesnichestvo” forestry, 55°44′44.6″N, 048°47′29.2″E	stump of deciduous tree
Russulales	*Hericiaceae*	*Hericium coralloides* (Scop.) Pers., LE-BIN 3594	OM033735	08-Aug-2015	Russia, Bryansk Region, “Bryansk Forest” Nature Reserve, 52º26′54″N, 033º51′60″E	fallen wood of aspen
